# Growth Characteristics and Stock Assessment of *Sebastes schlegelii* in the Northern Yellow Sea off Liaoning Province, China

**DOI:** 10.3390/ani16182822

**Published:** 2026-09-08

**Authors:** Yikai Lan, Zengqiang Yin, Lin Zhang, Quan Yu, Jiahang Wei, Fan Du, Lei Chen, Jun Yang, Tao Tian, Hongmei Li

**Affiliations:** 1 College of Marine Science and Environment, Dalian Ocean University, Dalian 116023, China; lanyikai2002@163.com (Y.L.); 18842508412@163.com (L.Z.); 18040290235@163.com (J.W.); dfanyj@163.com (F.D.); chenlei@dlou.edu.cn (L.C.); yangj@dlou.edu.cn (J.Y.); 2 College of Fisheries and Life Science, Dalian Ocean University, Dalian 116023, China; 18957231585@163.com (Q.Y.); tian2007@dlou.edu.cn (T.T.); 3 Shanghai Society of Oceanology and Limnology, East China Sea Ecology Center, Ministry of Natural Resources, Shanghai 200062, China; limailg@126.com

**Keywords:** *Sebastes schlegelii*, growth characteristics, current status of resources, northern Yellow Sea

## Abstract

*Sebastes schlegelii* is a commercially significant species in the northern Yellow Sea. Recently, *Sebastes schlegelii* has shown a reduced stock density and a shift toward smaller body sizes in the northern Yellow Sea area off Liaoning Province. To comprehend the population dynamics and to formulate management tactics, we established a mathematical formula describing the length–weight relationship, as well as the corresponding growth equations for the body length and body weight. The biomass of *Sebastes schlegelii* in the northern Yellow Sea area off Liaoning Province was evaluated using biological survey data from 2019 to 2022. The findings reveal that the length–weight formula is expressed as *W* = 4.98 × 10^−5^*L*^2.8878^ (*R*^2^ = 0.9108), with *L*_∞_ = 407.6 mm, *W*_∞_ = 1717.04 g, *K* = 0.21 a^−1^, and *t*_0_ = −0.65 a. The critical age was 3.34 years, and the stock biomass was 13,141.35 tonnes. The current exploitation rate of this resource is below the maximum yield, indicating sustainable utilization. A fishing closure period from April to June is considered suitable. A minimum landing size of 240.48 mm is recommended for *Sebastes schlegelii*.

## Simple Summary

*Sebastes schlegelii* is one of the key commercially important species in the northern Yellow Sea. In recent years, there has been a trend toward smaller body sizes and a decline in stock density. To understand the population dynamics of *Sebastes schlegelii* in the northern Yellow Sea area off Liaoning Province and to establish strategies for the rational utilization of this species, this study analyzed the growth characteristics and population dynamics of *Sebastes schlegelii* through a biological investigation and using social research data from 2019 to 2022. The study established a growth equation, determined the minimum landing size, assessed the stock size for this species in the area, and simulated changes in the egg production per recruit (EPR) and spawning biomass per recruit (SBR) under different management strategies. A rational fishing closure period was also determined. The findings of this study provide a reference for the sustainable utilization of the *Sebastes schlegelii* stock in the northern Yellow Sea area off Liaoning Province.

## 1. Introduction

Marine resources constitute an essential foundation for worldwide sustainable development. In 2015, the United Nations ratified the 2030 Agenda for Sustainable Development and the corresponding Sustainable Development Goals (SDGs) [1], specifically Goal 14, which aims to conserve and sustainably utilize oceans and marine resources by effectively regulating fishing, halting overfishing, and rehabilitating fish populations by 2030. In 2022, fisheries and aquaculture output reached an unprecedented 223.2 million tonnes, with the per capita aquatic animal food consumption estimated at 20.7 kg, representing around 15 percent of the animal protein supply. In various Asian and African nations, this percentage surpassed 50 percent. Despite the fact that capture fisheries’ output has mostly stabilized over the years, aquaculture has experienced a 6.6 percent increase since 2020 and presently constitutes over 57 percent of aquatic animal products intended for direct human consumption. Simultaneously, it is estimated that the fisheries and aquaculture industries directly employ around 62 million individuals and indirectly sustain the livelihoods of hundreds of millions through the value chain [2].

Nonetheless, the present condition of worldwide fishery resources faces various obstacles, such as illegal, unreported, and unregulated (IUU) fishing and insufficient coordination in the management of straddling fish stocks. Additionally, a considerable proportion of global fish stocks are currently classified as biologically unsustainable, indicating that catch levels have surpassed the maximum sustainable yield (MSY) [3]. However, Dr. Sumaila, a fisheries economist, argued that, if scientific and effective management measures were implemented for global fisheries, the ecological and economic benefits would far outweigh the management costs, demonstrating the long-term economic return of fisheries reconstruction [4].

*Sebastes schlegelii*, referred to as ‘black fish’, is a ray-finned species classified within the genus *Sebastes* of the Scorpaenidae family in the order Scorpaeniformes. The *Sebastes schlegelii* is a cold-temperate and demersal fish that lives in rocky reef environments with muddy or sandy substrates [5]. It grows relatively quickly, has delicious and nutritious meat, and possesses high economic value, making it one of the economically important fish species in the northern Yellow Sea. The northern Yellow Sea area of Liaoning Province has many islands, providing excellent habitats for *Sebastes schlegelii*. In recent years, human overfishing and marine environmental pollution have reduced the density of the *Sebastes schlegelii* stock, and miniaturization and youthfulness in populations have become increasingly severe, prompting local marine fisheries management authorities to focus on sustainable resource management [6].

Previous research on *Sebastes schlegelii* has primarily focused on aquaculture optimization and the effects of environmental changes on population distribution, for instance, investigations into the impacts of fluctuating water currents [7], diverse LED light spectra [8], and a sharp rise in water temperature [9] on the growth and metabolic processes of juvenile and adult fish, aimed at reducing the economic cost in commercial aquaculture and enhancing the survival rates of released juveniles in natural habitats. Zhang et al. explored the interactive effects of different environmental enrichment types and levels on the growth performance, behavioral performance, and stress-related physiological processes of this species [10]. Chen et al. employed species distribution models (SDMs) to forecast the prospective habitat distribution of *Sebastes schlegelii* in the marine area of China, South Korea, and Japan, clarifying the possible effects of climate change on future population dispersal and aiding in the execution of adaptive management strategies for this species [11].

The research on *Sebastes schlegelii* in China started relatively late, with the first relevant studies being reported in the late 1980s [12]. Thus far, Chinese scholars have performed comprehensive investigations into the biological attributes, behavioural characteristics, nutritional assessments [13], and captive breeding methodologies [14,15] of *Sebastes schlegelii*. For instance, Chen and colleagues employed a linear mixed-effects model to examine the spatiotemporal variability in the correlation between the body weight and body length of *Sebastes schlegelii* across three reef locations in Shandong, assessing the variations over different years, seasons, and marine zones [16]. Zhang et al. performed an initial investigation into the growth, mortality, and sustainable utilisation of *Sebastes schlegelii*, utilising frequency data regarding its body length, which suggests that the stock in reef regions experiences minimal exploitation and that catch rates could be enhanced by judiciously increasing fishing efforts [17]. Peng L et al. performed an investigation on the genetic variability and inherent population structure of 98 specimens of *Sebastes schlegelii* from three urban areas in northern China. According to the findings from neutrality assessments and the nucleotide mismatch frequency distribution, it was determined that the three regional populations of *Sebastes schlegelii* have not experienced significant population growth recently and should thus be preserved in situ as a complete entity [18]. There exists a scarcity of published studies regarding the stock assessment of *Sebastes schlegelii*; only a few studies evaluated the stock dynamics of *Sebastes schlegelii* in the vicinity of Zhangzi Island, Dalian [19], and no documented analyses have been identified concerning the stock dynamics of *Sebastes schlegelii* in the waters off Liaoning in the northern Yellow Sea. This study evaluates the growth traits and population dynamics of *Sebastes schlegelii* in the northern Yellow Sea area off Liaoning Province, utilising field survey data to aid in resource management.

## 2. Materials and Methods

### 2.1. Data Sources

**Figure 1:**
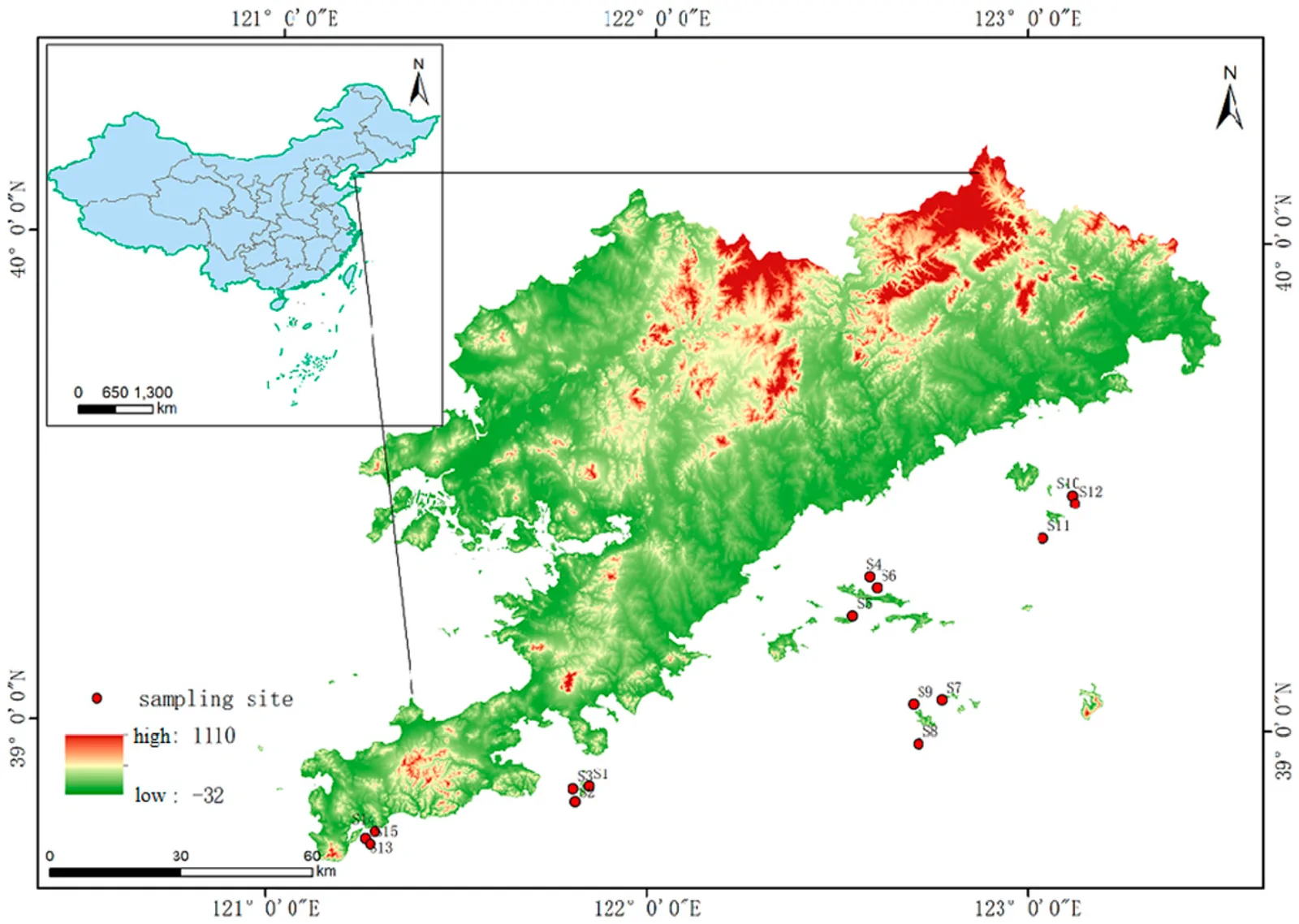
Stations for the resource survey of *Sebastes schlegelii*.

The data for this study were obtained from five field surveys of biological resources conducted in April 2019, September 2020, March 2021, and April and September 2022. Since *Sebastes schlegelii* prefers to inhabit nearshore rocky reef areas, sampling survey stations were set up according to the distribution characteristics of rocky reefs in the northern Yellow Sea, as shown in Figure 1, for the five islands along the coast of Liaoning Province: Sanshan Island, Wangjia Island, Zhangzi Island, Dachangshan Island, and Tahe Bay waters. The survey used a ground cage net (Wudi Jinhai Fishing Tackle Co., Ltd., Binzhou, China) with the main parameters of a mesh size of 25 mm, length of 13.0 m, width of 0.4 m, and height of 0.3 m. Sample acquisition and biological determination were conducted in compliance with the Specifications for Marine Fishery Resource Surveys (SC/T 9403-2012) [20]. Upon finishing the survey, the collected *Sebastes schlegelii* were promptly transferred to an insulated container (Guangzhou Aoxue Refrigeration Equipment Co., Ltd., Guangzhou, China) for refrigeration. The biological indicators of the *Sebastes schlegelii* include body length (accurate to 1 mm), body weight (accurate to 0.1 g), gender, and gonadal maturity.

### 2.2. Methods

#### 2.2.1. Methods for Studying Growth Characteristics

(1) Examining the composition of body length and body weight: Perform an analysis of covariance (ANCOVA) on body length and body weight composition for male and female fish. If there is a significant difference in ANCOVA, analyse the data by gender; if not, analyse the data as a whole.

(2) Formulating the equation that connects body length with body weight: The correlation between body length and body weight in *Sebastes schlegelii* is represented by a power function [21], articulated as follows:

*W_t_* = *a⋅L_t_^b^* (1)

where *W_t_* and *L_t_* denote body weight (g) and body length (mm) at age *t*, respectively; *a* signifies the condition factor parameter, while *b* indicates the power exponent.

(3) Estimating length and weight growth equations: The Von Bertalanffy growth function (VBGF) was applied to estimate the length and weight growth equations of *Sebastes schlegelii*:

*L_t_* = *L_∞_*[1 − *e^−K(t−t^*^0*)*^] (2)

*W_t_* = *W_∞_*[1 − *e^−K^^(^^t−t^*^0^*^)^*]^b^ (3)

where *L_∞_* and *W_∞_* denote the asymptotic length and asymptotic weight, respectively; *K* signifies the growth curvature; and *t*_0_ indicates the theoretical initial age. *L_∞_* and *K* were assessed utilizing the ELEFAN (Electronic Length Frequency Analysis I) tool within the FISAT II (version 1.2.2) software [22]; the theoretical initial age *t*_0_ can be derived through Pauly’s empirical formula [23]:

ln(−*t*_0_) = −0.3922 − 0.2752ln*L_∞_* − 1.038ln*K* (4)

The asymptotic weight (*W_∞_*) is then derived from Equation (5):

*W_∞_* = *a∙L_∞_^b^* (5)

(4) Determining the inflection age: The inflection age was defined as the age corresponding to the maximum growth rate and was estimated by setting the second derivatives of the length and weight growth equations of *Sebastes schlegelii* to zero [24], specifically d^2^*L_t_*/d*t*^2^ = 0 or d^2^*W_t_*/d*t*^2^ = 0.

#### 2.2.2. Methods for Studying Population Dynamics

(1) Estimating mortality and exploitation rate: The total mortality coefficient (*Z*) was estimated using the length-converted catch curve method, and the natural mortality coefficient (*M*) was obtained from Pauly’s empirical formula [25]:

lg*M* = −0.0066 − 0.279lg*L_∞_* + 0.6543lg*K* + 0.4634lg*T* (6)

In this context, T denotes where T is the annual average habitat water temperature; this study takes *T* = 18 °C.

The fishing mortality coefficient (*F*) is calculated by subtracting the natural mortality coefficient (*M*) from the total mortality coefficient (*Z*); the exploitation rate (*E*) is the fraction of overall mortality due to fishing, defined as *E* = *F*/*Z*.

(2) Estimating the critical age: The critical age represents the age at maximum cohort biomass under unfished conditions and corresponds to the point at which the relative growth rate of body mass equals the instantaneous natural mortality rate. It was calculated using Equation (7) [26]:

*T_c_* = [*kt*_0_ − ln*M* + ln(3*k* + *M*)]/*k* (7)

(3) Estimating the population number: The body-length-group analytical technique was employed to assess the population number of *Sebastes schlegelii* in the northern Yellow Sea area off Liaoning Province; the primary equation is outlined below [22]:

*N_t_* = *C_t_*⋅(*M + F_t_*)∕*F_t_* (8)

*C_i_* = *N_i+_*_∆*t*_∙(*F_t_*∕*M_t_*)∙(1 − *e*^−(^*^M+Ft^*^)∆*ti*^) (9)

*N_i_ = N_i+_*_∆*t*_∙*e^M+Fi^* (10)

where △*t_i_* = (*t_i+_*_1_ − *t_i_*), *t_i_* = *t*_0_ − (1/*K*)·ln(1 − *L_i_*/*L*_∞_), *C_t_* represents the catch at the maximum body length of *Sebastes schlegelii*; *M* signifies the natural mortality coefficient; *F_t_* is the fishing mortality coefficient for *Sebastes schlegelii* at its maximum body length; *N_i_* and *N_i+_*_△_*_t_* represent the population sizes of *Sebastes schlegelii* at ages *i* and *i +* △*t*, correspondingly; *C_i_* signifies the catch at age *i*; while *F_i_* indicates the fishing mortality rate for *Sebastes schlegelii* at age *i*.

(4) Egg Per Recruitment model (EPR) and Spawning Biomass Per Recruitment model (SBR): Per-recruit models are widely applied in fisheries management to assess the effects of fishing pressure on population reproductive potential. The EPR and SBR models provide quantitative measures of reproductive output and spawning stock biomass per recruit, allowing evaluation of the risk of recruitment overfishing. This study applied EPR and SBR analyses to assess the responses of *Sebastes schlegelii* reproductive potential to different fishing pressures and management strategies in the northern Yellow Sea off Liaoning Province, thereby providing a scientific basis for sustainable resource management. The equations for determining EPR and SBR are outlined below [27,28]:

(11) $$\mathrm{EPR}=\frac{\mathrm{EP}}{R}=\sum_{t=0}^{t_{\max}}\exp\left(-\left(\left(F\cdot S_{t}\cdot A_{t}\right)+M\right)t\right)\cdot P_{t}\cdot g_{t}\cdot e_{t}$$

(12) $$\mathrm{SBR}=\frac{\mathrm{SB}}{R}=\sum_{t=0}^{t_{\max}}\exp\left(-\left(\left(F\cdot S_{t}\cdot A_{t}\right)+M\right)t\right)\cdot a{\left(L_{t}\right)}^{b}\cdot G_{t}$$

where *EP* denotes the aggregate spawning output; *SB* signifies the cumulative spawning stock biomass; *g_t_* indicates the sex ratio of fish at age *t*; *e_t_* reflects the spawning output of fish at age *t*; *R* represents the recruitment rate, assumed to be 1; *P_t_* illustrates the fraction of fish at age *t* that have attained sexual maturity; *F* and *M* denote the coefficients for fishing and natural mortality, respectively; *a* and *b* signify the constants that correlate body length with body weight; *L_t_* indicates the mean total length at age *t* months; *t_max_* refers to the maximum age of *Sebastes schlegelii* within the sample; *A_t_* denotes if the Gregorian calendar month associated with age *t* is a closed season (0) or not (1); and the initial month t = 0 is designated as March, when *Sebastes schlegelii* larvae first emerge. The sexual maturity index *G_t_* and the gear selectivity index *S_t_* exhibit a ‘knife-edge’ [29] characteristic; hence, *G_t_* equals 0 when *t* is less than *t_m_* and 1 otherwise, whereas *S_t_* equals 0 when *t* is below *t_c_* and 1 when *t* meets or exceeds *t_c_*. In this context, *t_m_* signifies the age at which 50 percent sexual maturity is reached, while *t_c_* indicates the age when fishing begins.

## 3. Results

### 3.1. Growth Characteristics

#### 3.1.1. Body Length Composition and Body Weight Composition

**Figure 2:**
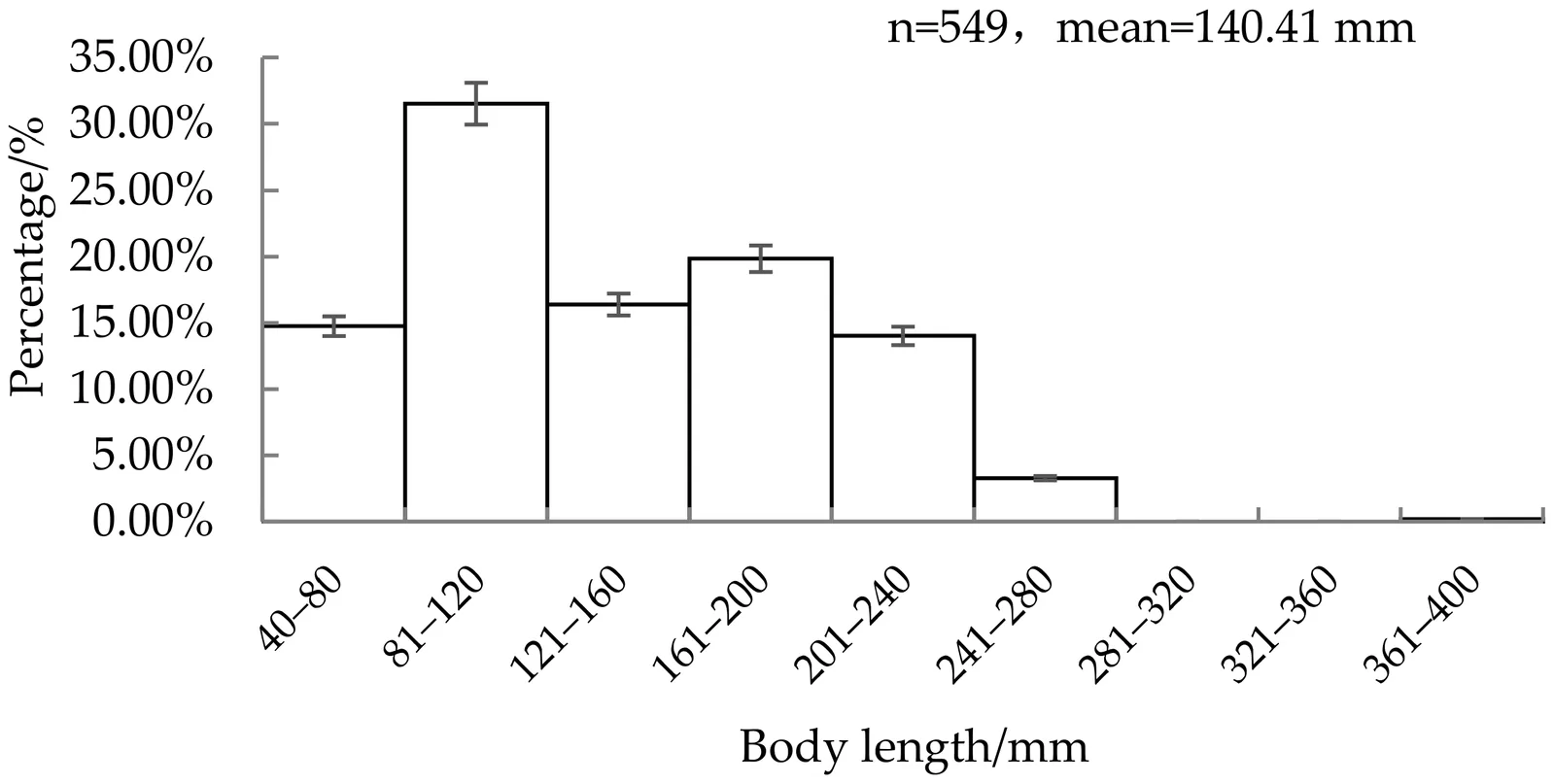
Length composition of *Sebastes schlegelii* in the northern Yellow Sea area off Liaoning Province.

**Figure 3:**
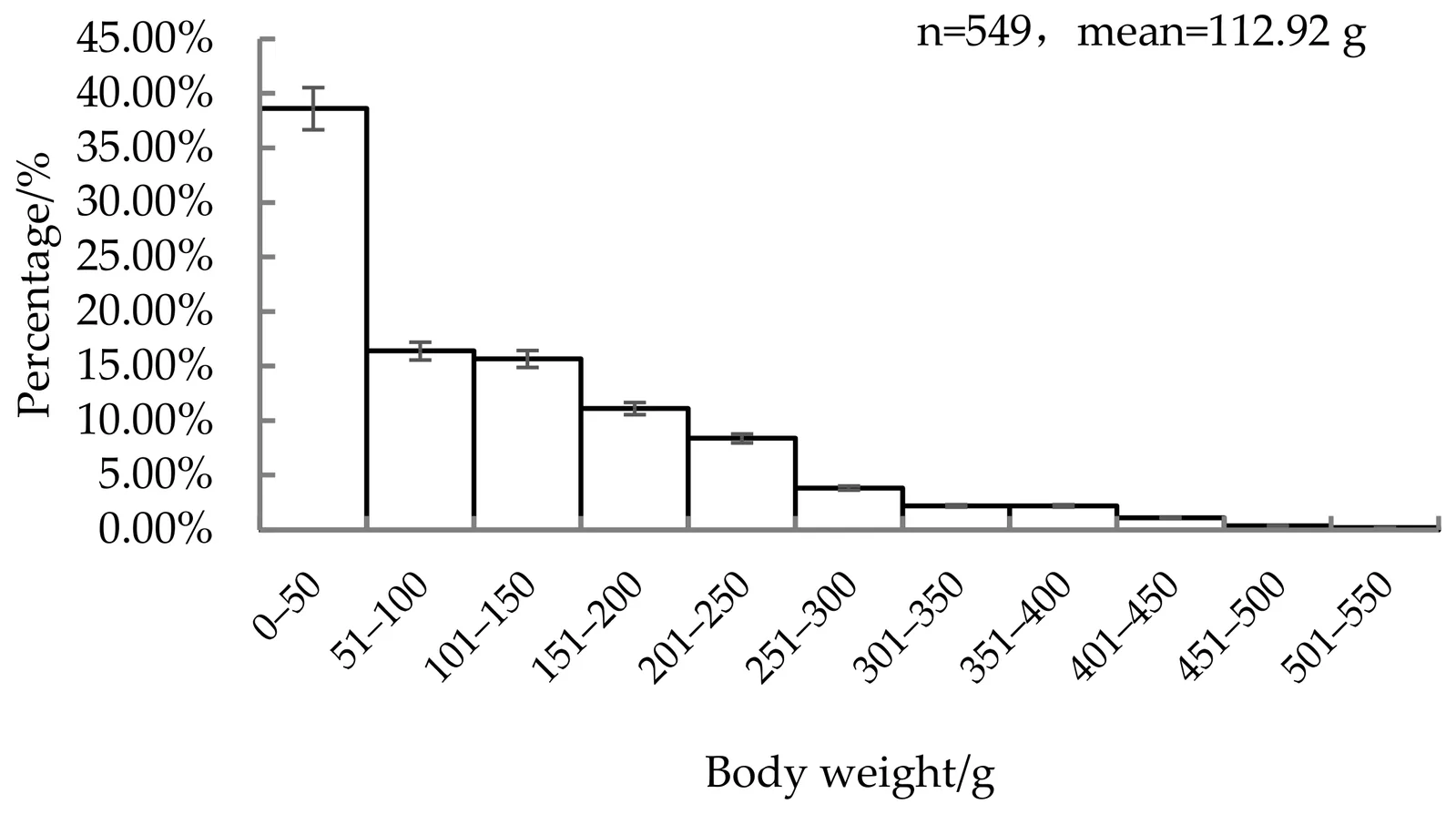
Weight composition of *Sebastes schlegelii* in the northern Yellow Sea area off Liaoning Province.

The analysis of covariance (ANCOVA) revealed no significant difference in body length composition between female and male *Sebastes schlegelii* in the northern Yellow Sea off Liaoning Province (*p* > 0.05; Table 1). Body length ranged from 41 to 385 mm, with a mean of 140.41 mm, while body weight ranged from 1.4 to 550 g, with a mean of 112.92 g. The most frequent length class was 81–120 mm, accounting for 31.51% of the individuals (Figure 2). In terms of body weight, the 1.4–50.04 g class was predominant, comprising 38.62% of the individuals (Figure 3).

#### 3.1.2. Equation for the Correlation Between Body Length and Body Weight

**Figure 4:**
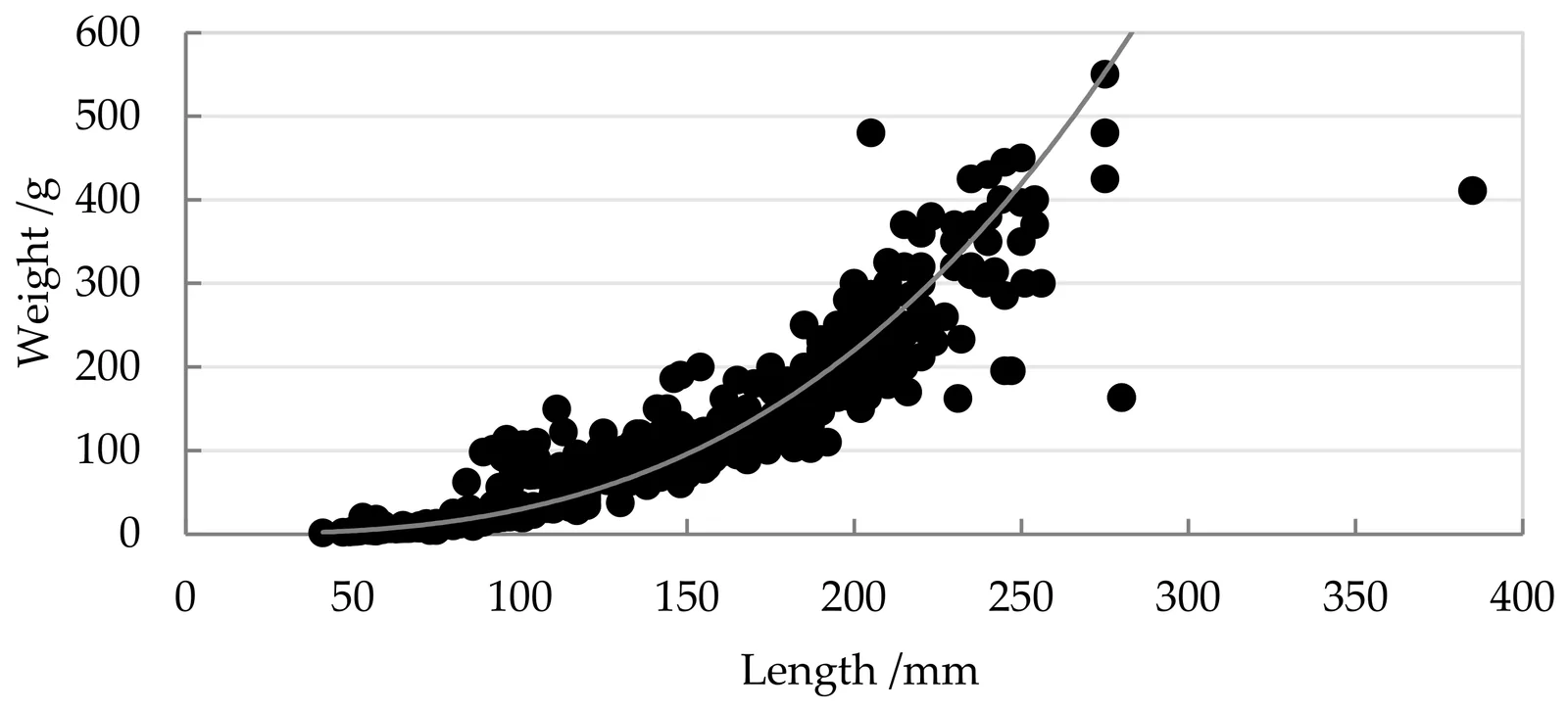
Body length–weight relationship curve of *Sebastes schlegelii* in the northern Yellow Sea area off Liaoning Province.

The analysis of covariance (ANCOVA) indicated that there was no statistically significant difference in growth between female and male *Sebastes schlegelii* specimens in the northern Yellow Sea area off Liaoning Province (*p* > 0.05). Applying a power function to their body length and weight (Figure 4) produced the subsequent equation: *W* = 4.98 × 10^−5^*L*^2.8878^ (*R*^2^ = 0.9108, *n* = 549).

#### 3.1.3. Growth Equation

**Figure 5:**
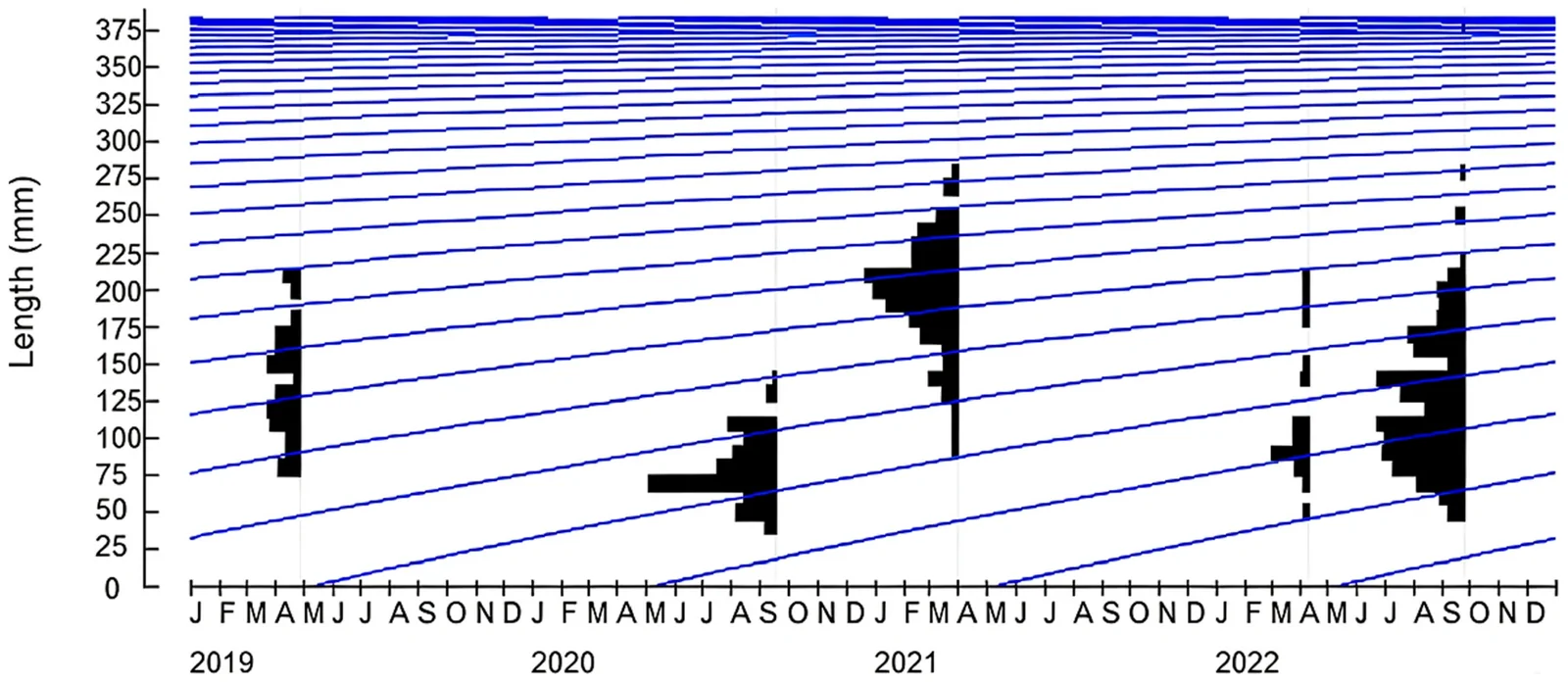
Body length distribution of *Sebastes schlegelii* in the northern Yellow Sea area off Liaoning Province from 2019 to 2022.

Body length frequency data were collected by categorising and tallying the body lengths of *Sebastes schlegelii* in the northern Yellow Sea area off Liaoning Province from 2019 to 2022 at intervals of 10 mm (Figure 5). Figure 5 illustrates that the length-frequency data bars connected by the same blue line represent the body length trends of fish from the same cohort, i.e., showing the modal length groups shifting across 2019 to 2022. The parameters of the von Bertalanffy growth equation were established using the ELEFAN I program in the FISAT II software as follows: *L_∞_* = 407.6 mm, *K* = 0.21 a^−1^, *W_∞_* = 1717.04 g, and *t*_0_ = −0.653 a. The optimal combination of asymptotic length (*L_∞_*) and growth coefficient (*K*) was determined via response surface analysis using the goodness-of-fit index Rn as the core evaluation indicator [22]. Through repeated fitting and verification processes, we demonstrate that the highest Rn value of 0.565 is achieved at *L_∞_* = 407.6 mm and *K* = 0.21 a^−1^, indicating this parameter set delivers the best-fitting growth model for the local target stock. The formulae for the increase in body length and body weight are *L_t_* = 407.6[1 − e^−0.21(*t*+0.653)^] and *W_t_* = 1717.04[1 − e^−0.21(*t*+0.653)^]^2.8878^, respectively.

#### 3.1.4. Age at Inflection Point

**Figure 6:**
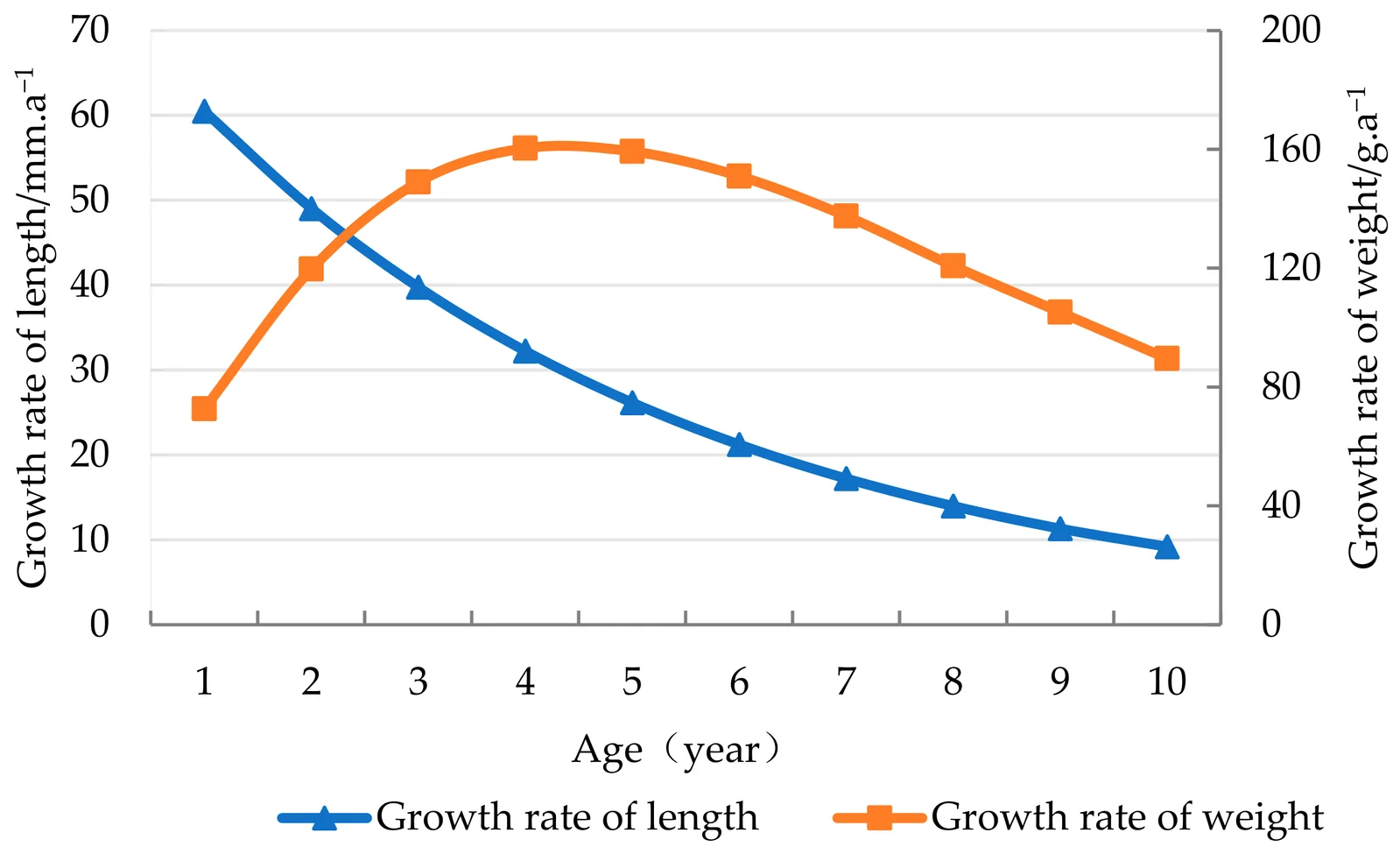
Growth rate curve of *Sebastes schlegelii* in the northern Yellow Sea area off Liaoning Province.

The initial derivative of the growth equations for the body length and weight of *Sebastes schlegelii* produces the subsequent growth rates: d*L_t_*/d*t* = 85.596e^−0.21(*t*+0.653)^ and d*W_t_*/d*t* = 1041.36e^−0.21(*t*+0.653)^[1 − e^−0.21(*t*+0.653)^]^1.8878^. The growth curves of length and weight against age for *Sebastes schlegelii* (Figure 6) indicated that the weight growth exhibited an increasing–decreasing pattern, with an inflection point, whereas the length growth declined continuously without an obvious inflection point. By solving d^2^*W_t_*/d*t*^2^ = 0, the inflection age was estimated as 4.397 years, corresponding to a body weight of 503.1 g and a body length of 266.57 mm.

### 3.2. Characteristics of Population Dynamics

#### 3.2.1. Mortality Rate and Development Rate

**Figure 7:**
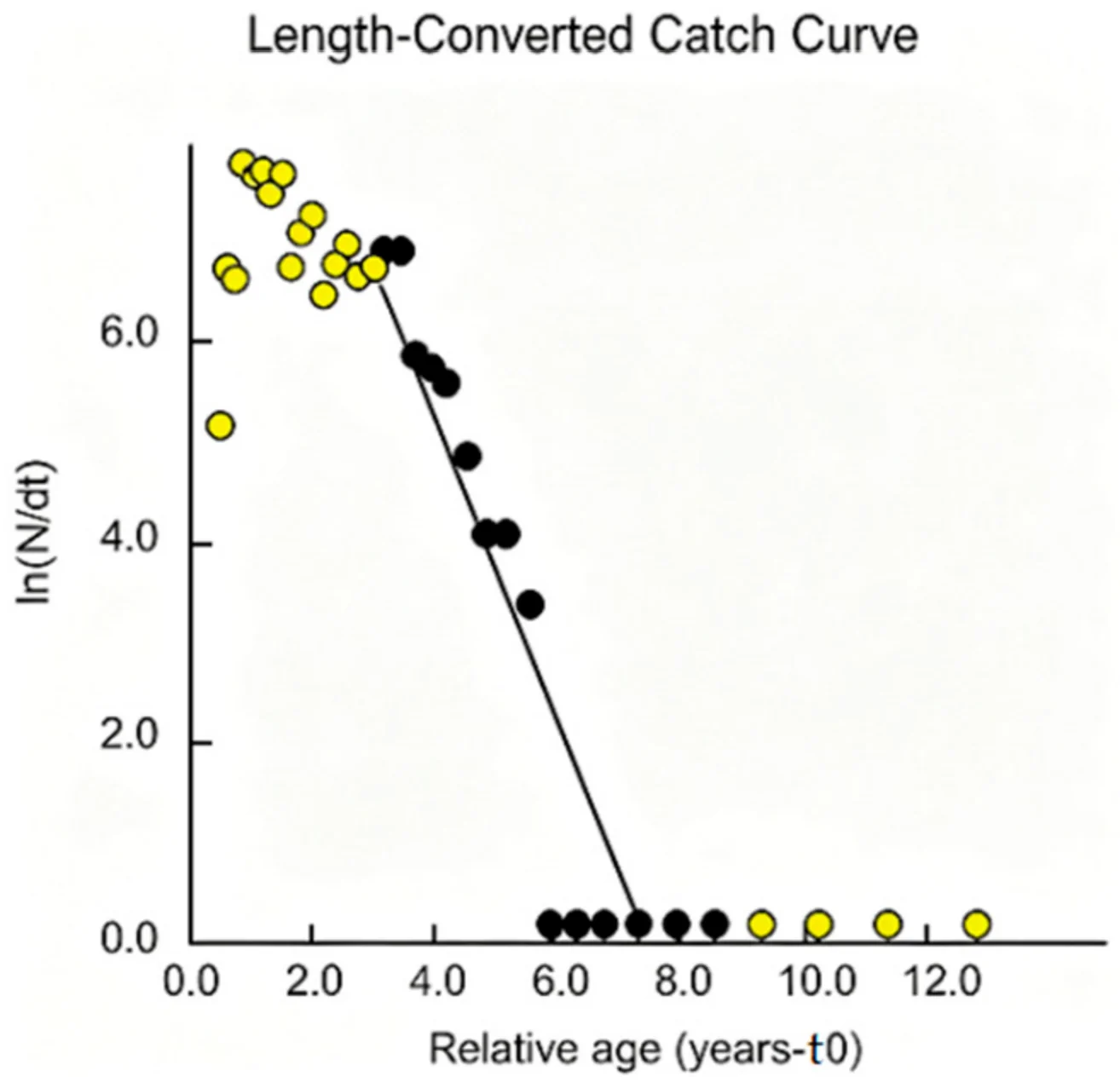
Catch conversion curve of body length of *Sebastes schlegelii* in the northern Yellow Sea area off Liaoning Province.

The overall mortality coefficient (*Z*) for *Sebastes schlegelii* was calculated from the length–catch conversion curve (Figure 7), specifically ln(*N*/△*t*) = −1.527*t* + 9.523 (correlation coefficient *R* = 0.9332); thus, *Z* = 1.53. Per Equation (6), the natural mortality coefficient (*M*) is 0.4807; the fishing mortality coefficient is calculated as *F* = *Z* – *M* = 1.0493; and the exploitation rate *E* for *Sebastes schlegelii* in the northern Yellow Sea area off Liaoning Province is *E* = *F/Z* = 0.6858.

#### 3.2.2. Critical Age

According to Equation (7), the critical age *T_c_* of *Sebastes schlegelii* in the northern Yellow Sea area off Liaoning Province is determined to be 3.3351 years; inserting this value into the growth equation results in a critical body length and weight of 231.19 mm and 333.1 g, respectively.

#### 3.2.3. Resource Estimation

**Figure 8:**
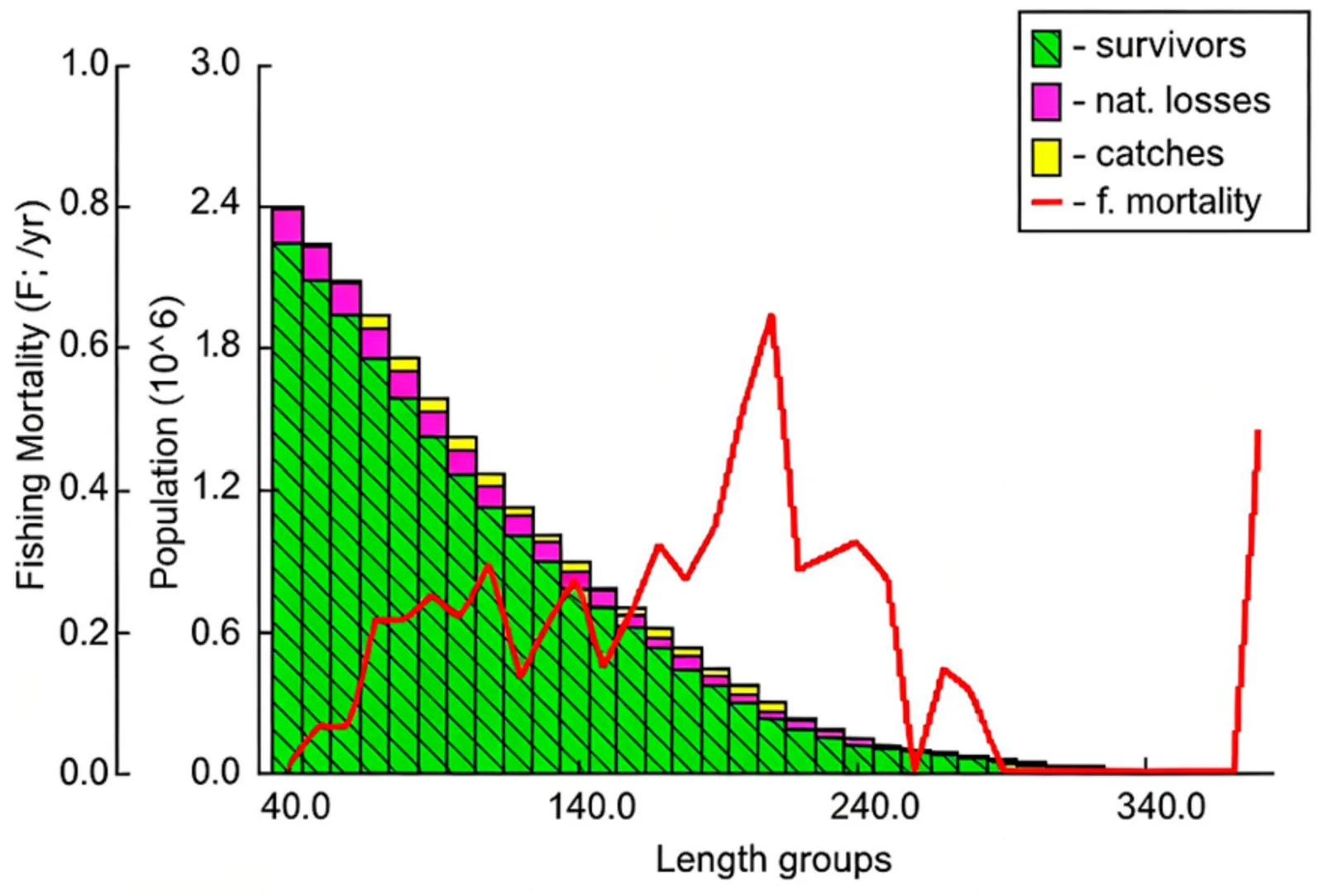
Resource biomass assessment of *Sebastes schlegelii* in the northern Yellow Sea area off Liaoning Province.

The exploitation rate for *Sebastes schlegelii* at its maximum body length is typically regarded as 50% [29]; hence, the fishing mortality coefficient for this length class is established at *F_t_* = *M* = 0.4807. By integrating this with the length-class analysis technique and employing FISAT II to assess the stock size (Figure 8), the biomass of the *Sebastes schlegelii* population in the northern Yellow Sea area off Liaoning Province was determined to be 13,141.35 tonnes.

#### 3.2.4. Current Status of Resource Utilisation

**Figure 9:**
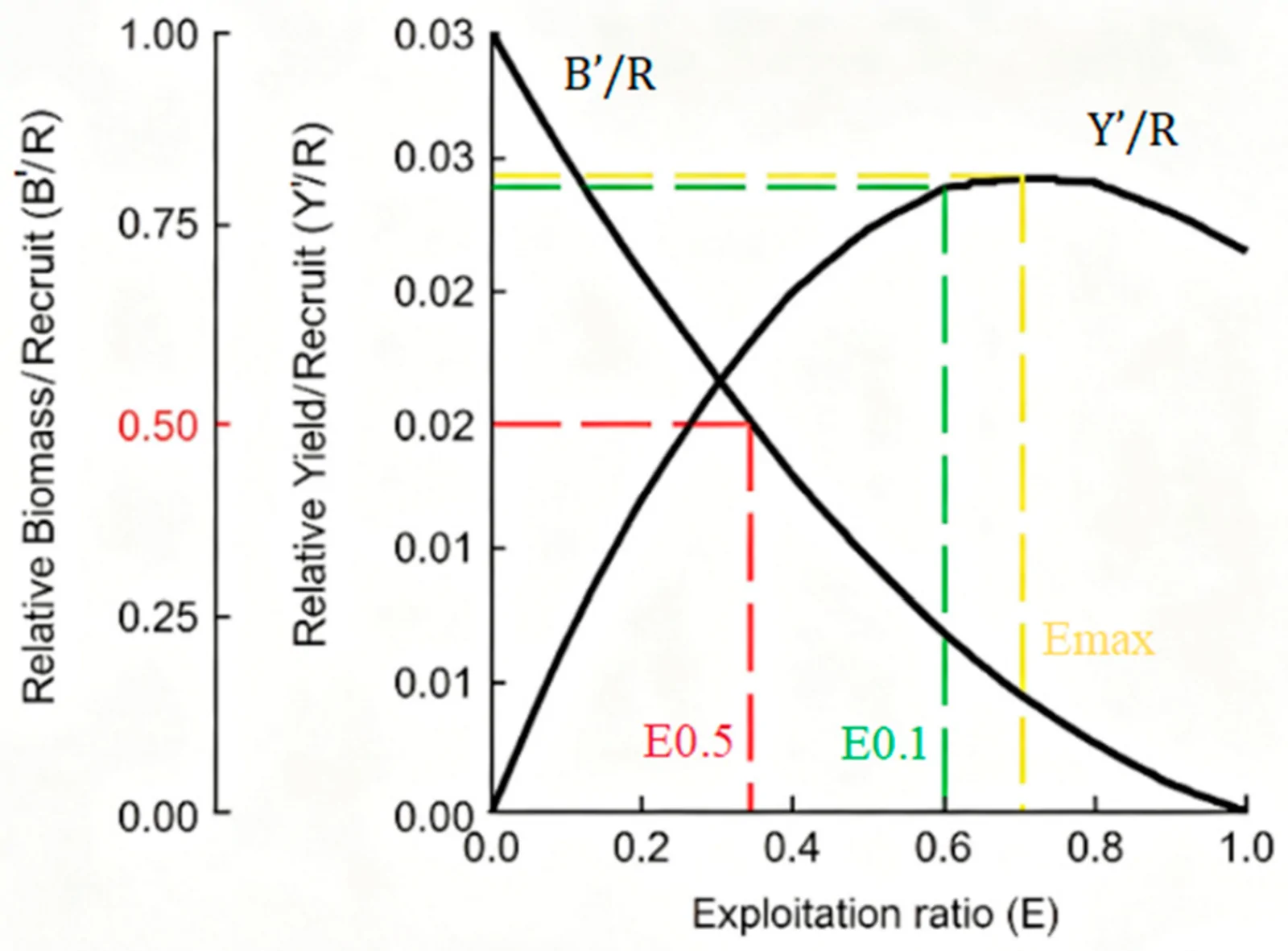
The relationship curve between relative yield per recruit and relative biomass per recruit and exploitation ratio of *Sebastes schlegelii* in the northern Yellow Sea area off Liaoning Province.

**Figure 10:**
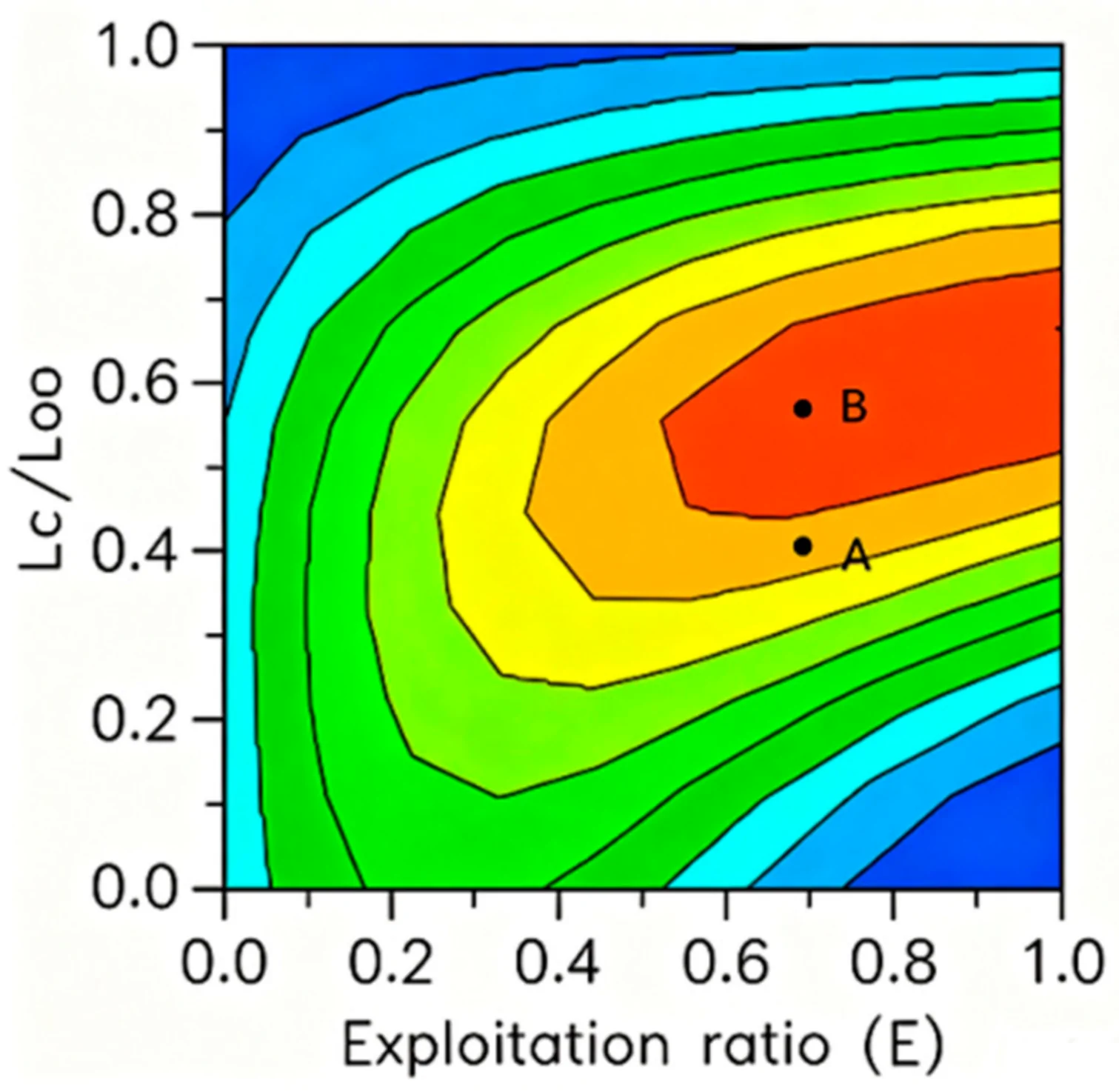
The relationship graph between relative yield per recruit and exploitation ratio and catchable size of *Sebastes schlegelii* in the northern Yellow Sea area off Liaoning Province.

The selectivity curve for fishing gear indicates that the frequency distribution of capture sizes for *Sebastes schlegelii* enables the calculation of the opening size *L_c_* as 172.33 mm, with the exploitation rate *E*, *L_c_*/*L_∞_*, and *M/K* being 0.6858, 0.423, and 2.289, correspondingly. The FISAT II software enables the assessment of the correlations between the relative yield per unit replenishment (*Y’/R*) and biomass (*B’/R*), along with the exploitation rate *E* (Figure 9), as well as the association between the relative yield per unit replenishment (*Y’/R*) and *L_c_/L_∞_*, and the exploitation rate *E* (Figure 10).

Figure 9 illustrates that the catch per unit of recruitment peaks with rising exploitation rates before thereafter declining, whereas the biomass per unit of recruitment persistently diminishes as the exploitation rates escalate. The present exploitation rate *E* (0.6858) for *Sebastes schlegelii* in the northern Yellow Sea area off Liaoning Province surpasses the optimal yield exploitation rate *E*_0.1_ (0.604) and the maximum sustainable yield exploitation rate *E*_0.5_ (0.343); yet, it has not surpassed the maximum yield exploitation rate *E*_max_ (0.706), indicating that the stock is being utilised sustainably; nevertheless, if suitable management strategies are not implemented swiftly, the *Sebastes schlegelii* population in the northern Yellow Sea area off Liaoning Province could soon face over-exploitation.

In Figure 10, point A denotes the present resource utilisation level, whereas point B signifies the point at which the catch is maximised in relation to the unit replenishment rate given the existing exploitation rate. As a result, the present resource utilisation level for *Sebastes schlegelii* in the northern Yellow Sea area off Liaoning Province (specifically, black point A (*L_c_/L_∞_* = 0.423, *E* = 0.6858)) indicates a notably low *L_c_/L_∞_* ratio. To enhance the yield, it is essential to elevate the *L_c_/L_∞_* ratio, specifically by augmenting the minimum catch size *L_c_*. The optimal yield is attained when *L_c_* equals 240.48 mm.

#### 3.2.5. Simulation of Fishing Moratorium Strategies

The summer fishing moratorium is an important management measure for conserving fishery resources by providing protection for spawning adults and feeding juveniles during critical life-history stages, thereby reducing fishing pressure and supporting population recovery. To improve the conservation of commercially important rocky-reef fishes such as *Sebastes schlegelii*, the timing of fishing moratoriums should be aligned with their reproductive cycles, particularly the periods of adult spawning and juvenile growth. This study applied EPR and SBR models to evaluate the changes in the reproductive potential and population recovery capacity of *Sebastes schlegelii* under different fishing moratorium strategies. The results provide a scientific basis for refining seasonal fishing closures tailored to the current population status of *S. schlegelii* in the northern Yellow Sea off Liaoning Province, supporting the sustainable management of this species. The breeding period for *Sebastes schlegelii* occurs annually from March to June, with heightened activity primarily in April and May; certain early or late-maturing specimens may deliver offspring as soon as late March or as late as early June [12,30]. The impacts of the subsequent four distinct management strategies on the EPR and SBR values were modelled according to these characteristics; the pertinent parameter values are displayed in Table 2.

**Figure 11:**
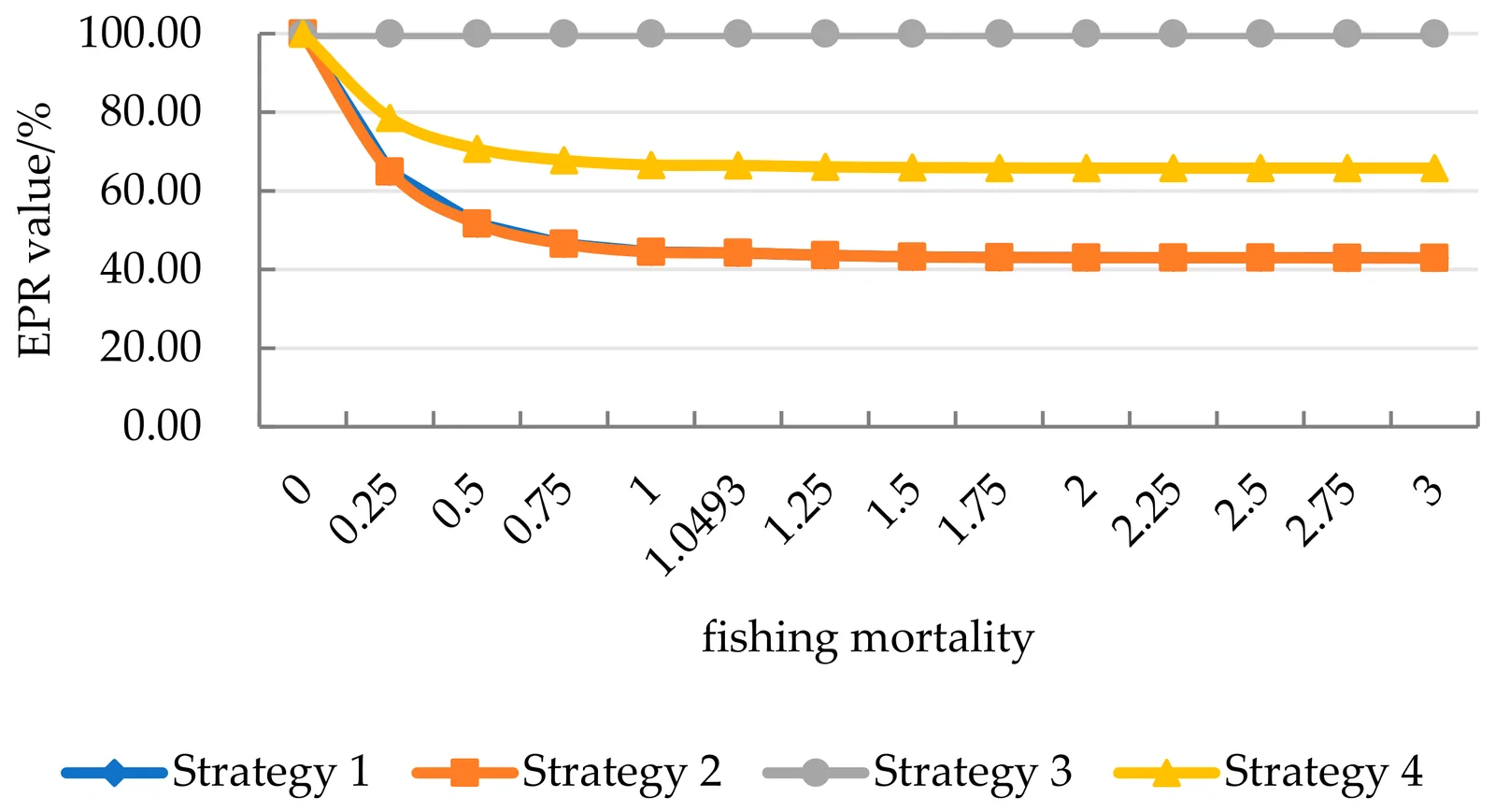
Change curve of EPR value with fishing death coefficient in *Sebastes schlegelii* under different strategies.

**Figure 12:**
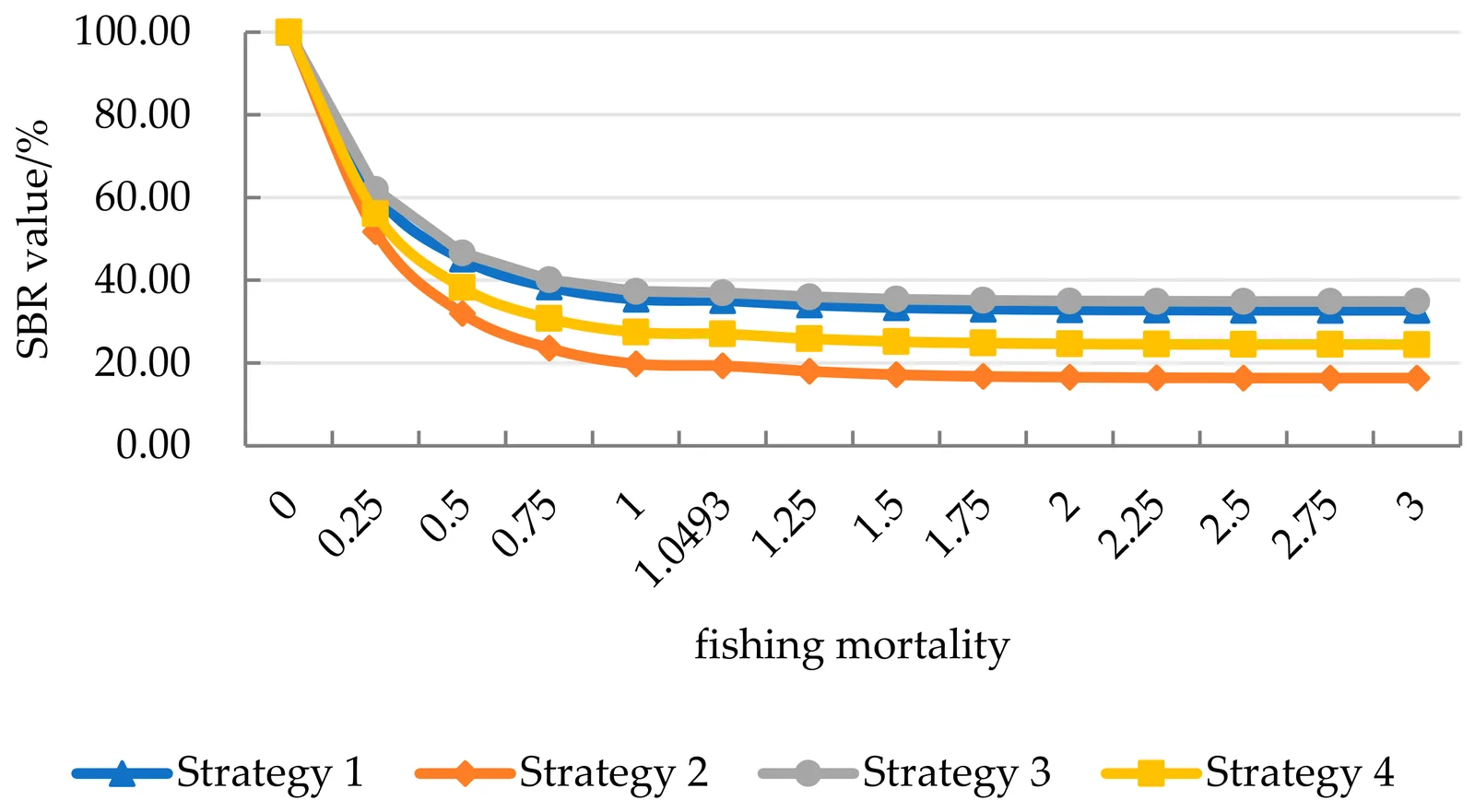
Change curve of SBR value with fishing death coefficient in *Sebastes schlegelii* under different strategies.

Based on Equations (11) and (12), the relative values of EPR and SBR under different instantaneous fishing mortality rates were calculated using the unfished conditions as reference and are presented in Table 3. The effects of different simulation strategies on EPR and SBR are illustrated in Figure 11 and Figure 12.

(1) Strategy 1: The fishing moratorium runs from May to August, lasting four months; this is the current fishing moratorium arrangement.

(2) Strategy 2: The fishing moratorium runs from May to June, lasting two months.

(3) Strategy 3: The fishing moratorium runs from March to June, lasting four months; this coincides with the spawning season for *Sebastes schlegelii*.

(4) Strategy 4: The fishing moratorium runs from April to June, lasting three months.

The data illustrated in Figure 11 and Figure 12 indicate that the EPR and SBR values diminish consistently as the fishing mortality coefficient *F* escalates, with the rate of decline progressively decelerating as *F* nears 1. Under the prevailing fishing intensity, the implementation of Strategy 1 within the existing fishing moratorium management framework results in the EPR value representing 44.23 percent of the theoretical maximum value in an undisturbed condition, while the SBR value constitutes 35.07 percent of the theoretical maximum value in an undisturbed state; upon the implementation of Strategy 2, the EPR value, similar to Strategy 1, constitutes 44.23 percent of the theoretical maximum value in the undisturbed condition, whereas the SBR value represents 19.35 percent of the theoretical maximum value in the undisturbed condition; in Strategy 3, the EPR value signifies the entirety of the theoretical maximum value in underdeveloped conditions, whereas the SBR value accounts for 36.97 percent of the theoretical maximum value in undeveloped situations; and, under Strategy 4, the EPR value accounts for 66.49 percent of the theoretical maximum value in undeveloped conditions, whereas the SBR value constitutes 27.04 percent of the theoretical maximum value in undeveloped situations.

## 4. Discussion

### 4.1. Growth Characteristics Parameters

The length and mass are key indicators of fish growth and population status. Survey data of *Sebastes schlegelii* collected from the northern Yellow Sea off Liaoning Province during 2019–2022 revealed a length range of 41–385 mm, with most individuals concentrated in the 81–120 mm group. The mean length (140.41 mm) was markedly lower than the reported length at first sexual maturity (*L_m_* = 212.5 mm) [31], suggesting that a large proportion of individuals had not reached reproductive maturity. It can be seen that the percentage of juvenile *Sebastes schlegelii* in this region is increasing, and the inclination toward smaller specimens is becoming more evident.

In the response surface analysis of ELEFAN I implemented in FISAT II, we constrained the scoring values within the range above 90% of the maximum score, resulting in confidence intervals of 387.22–415.75 mm and 0.15–0.23 for the biological parameters *L_∞_* and *K*, respectively. Furthermore, as shown in Table 2, the relevant biological parameters of *Sebastes schlegelii* calculated in this study, including *L_∞_* = 407.6 mm and *K* = 0.21 a^−1^, are relatively close to the previously published research results of other scholars on the same species in the identical sea area. The estimated asymptotic length *L_∞_* and growth coefficient *K* values from their study are 412.5 mm and 0.21 a^−1^ [19], as well as 450 mm and 0.31 a^−1^ [32], respectively. This numerical proximity effectively confirms the rationality of our parameter calculation, and further enhances the reliability of subsequent downstream related parameters and stock biomass estimation results.

*e^−K^* serves as an indicator for evaluating how well the Von Bertalanffy growth equation corresponds to fish growth; an *e^−K^* value of less than 1 signifies that the equation offers an accurate representation of fish growth [33]. The computations presented in this manuscript demonstrate that *e^−K^* = 0.81 < 1, suggesting that this growth model accurately represents the growth of *Sebastes schlegelii*. The age at which the weight inflection point occurs, as derived from the growth equation, signifies the moment when the absolute growth rate of the fish body weight starts to decrease; hence, fish stocks ought to be harvested post this inflection age whenever feasible [34,35]. This research determined that the body length and weight at the inflection point age of *Sebastes schlegelii* are 266.57 mm and 503.1 g, respectively. The mean body length and weight of *Sebastes schlegelii* in the catch were 140.41 mm and 112.92 g, respectively, both of which were less than the inflection point for body length and weight; this suggests that most individuals in the catch were captured prior to attaining their peak growth rate.

### 4.2. Current Status of Resource Utilisation

The exploitation rate may act as a benchmark for evaluating the condition of fisheries resource utilisation [36]. Mehanna posits that, on the graph depicting the relationship between the yield and exploitation rate for a specific recruitment unit, a situation where the exploitation rate *E* is below the maximum yield exploitation rate *E*_max_ indicates sustainable resource use; conversely, when *E* surpasses *E*_max_, it signifies the overexploitation of the resource [37]. At the present body length (*L_c_*) of 172.33 mm where fishing begins, the *E*_max_ for *Sebastes schlegelii* in the northern Yellow Sea area off Liaoning Province is 0.706, while the exploitation rate is 0.6858, which is less than *E*_max_. These findings suggest that the stock is not currently overfished; however, continued fishing pressure without appropriate management measures may increase the risk of overexploitation for the *Sebastes schlegelii* population in the northern Yellow Sea off Liaoning Province. Provided that the exploitation rate is stable, when *Y’/R* attains its peak, *L_c_/L_∞_* equals 0.59, and the requisite minimum landing size is 240.48 mm. The existing minimum landing size in the northern Yellow Sea area off Liaoning Province is 172.33 mm, considerably less than 240.48 mm, suggesting a notable inclination toward smaller specimens of *Sebastes schlegelii* in these waters. Niu Keru et al. discovered that the body length and weight of *Sebastes schlegelii* captured near Zhangzi Island in the northern Yellow Sea were markedly less than those at their inflection point age [32]; thus, it is plausible that *Sebastes schlegelii* in this region were being harvested during a period of accelerated growth. The observed decline in body size may be related to the prolonged and intensive fishing pressure on *Sebastes schlegelii* populations, especially the harvesting of individuals before reaching sexual maturity. Such selective pressure could promote the prevalence of smaller-sized and earlier-maturing individuals in the spawning population over time. As a result, this has markedly enhanced the probability of diminutive people transmitting their genetic material to their progeny, propelling the entire population to progressively adapt toward reduced body dimensions.

Biological reference points are essential fundamental quantitative metrics in contemporary fisheries resource management frameworks. This notion was initially introduced by Schaefer and is often prominent in traditional analyses of fisheries population dynamics [38]. The fundamental principle involves creating a measurable evaluation system grounded in population biology traits, utilised to precisely ascertain if the exploitation condition of fishery resources remains within sustainable thresholds; the choice of biological reference points is dictated by modelling. The temporal mismatch between spawning and fishing activities may allow the *Sebastes schlegelii* stock to sustain a higher exploitation level than the current estimates suggest. Given the observed decrease in size at maturity and the absence of clear evidence of stock collapse in the northern Yellow Sea off Liaoning Province, the fishing mortality levels corresponding to 25% EPR and 50% SBR are suggested as precautionary threshold and target reference points, respectively, for fisheries management. In EPR and SBR models, these are, specifically, *F*_25%EPR_, *F*_25%SBR_, *F*_50%EPR_, and *F*_50%SBR_ [39,40].

According to Figure 11 and Figure 12, under the current fishing regime, the EPR value under Strategy 1 represents only 44.23% of the unfished level, suggesting a potential reduction in the reproductive capacity of the *Sebastes schlegelii* population in the northern Yellow Sea off Liaoning Province. The comparison of alternative fishing strategies suggests that, under the same fishing mortality (F), EPR is primarily affected by the extent of overlap between the fishing moratorium period and the spawning season of *Sebastes schlegelii*. Strategy 3 showed the highest simulated EPR value because its closure period fully overlapped with the spawning season, maximizing protection during reproduction. Strategy 4 showed an intermediate effect, as it overlapped with most of the spawning period but was less effective than Strategy 3. Although Strategies 1 and 2 both protected the stock during part of the spawning season, Strategy 1 provided a longer overall recovery period, which may contribute to differences in reproductive potential. Figure 12 and Table 3 indicate that, under the same fishing mortality (F), SBR was positively related to the duration of the fishing moratorium, suggesting that longer closures may enhance spawning stock recovery. Although Strategies 1 and 3 both lasted four months, Strategy 3 showed the highest SBR value due to its greater overlap with the reproductive period, followed by Strategy 1, Strategy 4, and Strategy 2.

According to Table 3, EPR and SBR values below 25% suggest that spawning stock biomass may be insufficient to maintain stable recruitment [29]. In this case, the population recruitment will be chronically lower than the average level, which further leads to the decline in population resources. This situation is a typical case of recruitment overfishing. When the fishing mortality coefficient F exceeds the fishing mortality coefficient corresponding to the maximum sustainable yield, this type of overfishing is defined as growth overfishing. At this point, the fishery faces problems such as a first capture age that is too low or excessive fishing intensity, and a large number of immature juveniles are captured in advance, which directly leads to a significant decline in the yield per generation.

In this study, the exploitation rate at maximum sustainable yield (*E*_0.5_) of the *Sebastes schlegelii* fishery is calculated to be 0.343, and the corresponding fishing mortality coefficient at the maximum sustainable yield level is 0.525. Combined with the results in Table 3, the actual fishing mortality coefficient *F* under the four current management strategies all reaches 1.0493, which is significantly higher than the threshold of 0.525. Therefore, all of them are in the state of growth overfishing. However, given the biological characteristics of *Sebastes schlegelii*, such as the high fecundity and strong recruitment potential, moderate fishing pressure under appropriate management measures may be compatible with sustainable resource utilization. Under the current fishing intensity, Strategies 3 and 4 showed potential for improving the reproductive status of the *S. schlegelii* population in the northern Yellow Sea off Liaoning Province. Considering both conservation objectives and socioeconomic factors, including the maintenance of spawning stock biomass, sustainable utilization, and fishermen’s livelihoods, Strategy 4 (April–June fishing moratorium) may represent a more balanced management option for the future exploitation of *S. schlegelii* resources. Further analysis suggests that, under Strategy 4, maintaining fishing mortality (F) at levels below 1.5 could help reduce the risk of growth overfishing. This strategy may provide an effective balance between conserving the *Sebastes schlegelii* population and reducing the economic impacts on fisheries, while offering insights for the design of seasonal fishing moratoriums in other regions.

### 4.3. Study Limitations

Although this study provided insights into the population dynamics and management strategies of *Sebastes schlegelii* in the northern Yellow Sea off Liaoning Province, several limitations should be considered when interpreting these findings.

First, limitations related to the survey design should be considered. The seasonal fishing closure prevented continuous sampling throughout the entire reproductive period, and the absence of samples during some months may have increased the uncertainty in the estimation of growth parameters using ELEFAN. Furthermore, the relatively limited sample size and spatial coverage may have affected the representativeness of the estimated population parameters. And this study uses length-frequency data to estimate the growth parameters (*L_∞_* and *K*) and total mortality coefficient, under the premise that recruitment and total mortality remain constant. The assumption is that the length-frequency distribution of the sampled population is identical to that of a single cohort. Therefore, there may be a certain deviation between the estimated growth parameters (*L_∞_* and *K*), total mortality coefficient, and their true values, which will bring certain impacts on the results of this study. Second, the use of bottom cage nets as the main sampling gear may have resulted in size-selective sampling, with a limited representation of very small individuals and larger individuals inhabiting deeper reef habitats. Such a sampling bias may have influenced the observed length and weight distributions. Third, the relatively small number of sampling sites and uneven spatial coverage across the northern Yellow Sea off Liaoning Province may have limited the representativeness of the population parameters estimated in this study.

However, the samples used in this study were all collected from key fishing grounds of the *Sebastes schlegelii* in the northern Yellow Sea area off Liaoning Province, and the data collected covered multiple months before and after the fishing closed period, while also attempting to encompass all four seasons of the year: March (the transition period from late winter to early spring), April (spring), and September (the end of the fishing closed period, transitioning from late summer to early autumn). The continuous length-frequency distribution indicates that these samples can represent the captured population. The key biological parameters such as body length, weight, and sex ratio obtained from the samples are similar to the long-term historical survey data of this sea area. Therefore, the conclusions of this study still possess sufficient representativeness and practical value, providing reliable scientific evidence for formulating targeted fishery management measures for the *Sebastes schlegelii* in the northern Yellow Sea area off Liaoning Province. To further improve the accuracy of the relevant parameters, we will expand the sampling range and increase the number of gear types and survey stations in subsequent studies to optimize the research results.

## 5. Conclusions

(1) In the northern Yellow Sea area off Liaoning Province, the average body length of *Sebastes schlegelii* was 140.41 mm, which is significantly smaller than the body length at first sexual maturity (212.5 mm) [31]; the population is characterised by a severe trend toward younger age classes;

(2) The present exploitation rate of *Sebastes schlegelii* in the northern Yellow Sea area off Liaoning Province considerably exceeds the rates linked to the optimal yield and maximum sustainable yield, although it is marginally lower than the rate corresponding to the maximum yield. This suggests that the stock is not now overfished; nonetheless, without timely management interventions, the *Sebastes schlegelii* stock in this region may soon become overexploited.

(3) The biomass of the *Sebastes schlegelii* stock in the northern Yellow Sea off Liaoning Province was estimated at 13,141.35 tonnes. Although the current fishing moratorium partially overlaps with the spawning season, the estimated EPR and SBR values represented only 44.23% and 35.07% of the unfished levels, respectively. Both values were below the target reference points, indicating that the reproductive capacity of the stock may not yet have recovered to an optimal level.

(4) Based on the estimated stock biomass and growth characteristics, a minimum landing size ranging from 231.19 to 266.57 mm could be considered for *Sebastes schlegelii* in the northern Yellow Sea off Liaoning Province. A minimum catch size of 240.48 mm may maximize the yield under the current model assumptions. Furthermore, a fishing moratorium from April to June may serve as a potential management option for future fisheries regulation.

## Figures and Tables

**Table 1:** ANCOVA results for sex-based growth differences in *Sebastes schlegelii.*

Source	F	*p*	Conclusion
Gender (controlling for Weight)	0.499	0.481	Not significant (*p* > 0.05 ^1^)
Gender (controlling for Length)	2.184	0.141	Not significant (*p* > 0.05)
Wilks’ Λ	1.354	0.260	Not significant

^1^ Note: Significance level was defined as α = 0.05, and no significant difference between groups was detected after covariate adjustment (*p* > 0.05).

**Table 2:** EPR and SBR model parameters.

Parameter	Value
a	4.98 × 10^−5^
b	2.8878
L_∞_/mm	407.6
*K*/a^−1^	0.21
*t*_0_/a	−0.653
*F*	1.0493
*M*	0.4807
*T_max_*/a	5
Body length at 50% sexual maturity/mm	212.5
Body length at first fishing (L_c_)/mm	172.33

**Table 3:** Changes in EPR and SBR values with fishing mortality coefficient under different strategies of *Sebastes schlegelii.*

Fishing Mortality (*F*)	Strategy 1		Strategy 2		Strategy 3		Strategy 4	
	EPR Value/%	SBR Value/%	EPR Value/%	SBR Value/%	EPR Value/%	SBR Value/%	EPR Value/%	SBR Value/%
0	100.00	100.00	100.00	100.00	100.00	100.00	100.00	100.00
0.25	64.95	60.94	64.95	51.75	100.00	61.96	78.57	56.07
0.5	51.69	45.12	51.69	32.03	100.00	46.60	70.75	38.29
0.75	46.53	38.41	46.53	23.58	100.00	40.13	67.78	30.76
1	44.46	35.43	44.46	19.81	100.00	37.30	66.62	27.44
1.0493	44.23	35.07	44.23	19.35	100.00	36.97	66.49	27.04
1.25	43.61	34.05	43.61	18.05	100.00	36.02	66.15	25.91
1.5	43.26	33.39	43.26	17.20	100.00	35.42	65.95	25.18
1.75	43.11	33.07	43.11	16.78	100.00	35.14	65.87	24.82
2	43.04	32.90	43.04	16.57	100.00	34.99	65.83	24.64
2.25	43.01	32.82	43.01	16.46	100.00	34.92	65.82	24.55
2.5	43.00	32.77	43.00	16.40	100.00	34.89	65.81	24.50
2.75	42.99	32.74	42.99	16.37	100.00	34.87	65.81	24.47
3	42.99	32.73	42.99	16.35	100.00	34.86	65.81	24.46

## Data Availability

The data of this study can be provided by the corresponding author upon request.

## Abbreviations

The following abbreviations are used in this manuscript:

IUU	Illegal, unreported and unregulated
MSY	Maximum Sustainable Yield
EPR	Egg Per Recruitment
SBR	Spawning Biomass Per Recruitment
